# Novel Inflammasome-Based Risk Score for Predicting Survival and Efficacy to Immunotherapy in Early-Stage Non-Small Cell Lung Cancer

**DOI:** 10.3390/biomedicines10071539

**Published:** 2022-06-28

**Authors:** Chih-Cheng Tsao, Hsin-Hung Wu, Ying-Fu Wang, Po-Chien Shen, Wen-Ting Wu, Huang-Yun Chen, Yang-Hong Dai

**Affiliations:** 1 Department of Radiation Oncology, Taitung MacKay Memorial Hospital, Taitung 950, Taiwan; kobeiori25@yahoo.com.tw; 2 Department of Pulmonary Medicine, Taitung MacKay Memorial Hospital, Taitung 950, Taiwan; a5809@mmh.org.tw; 3 Department of Radiation Oncology, Tri-Service General Hospital, National Defense Medical Center, Taipei 114, Taiwan; doc31006@mail.ndmctsgh.edu.tw (Y.-F.W.); 31235@mail.ndmctsgh.edu.tw (P.-C.S.); 4 Institute of Pathology and Parasitology, National Defense Medical Center, Taipei 114, Taiwan; vincent98115447@gmail.com; 5 Cancer and Hospice Care Center, Taitung MacKay Memorial Hospital, Taitung 950, Taiwan; a7676@mmh.org.tw

**Keywords:** early-stage NSCLC, immunotherapy, immune checkpoint inhibitor, inflammasome, IL1B, CASP-1, IL18, GSDMD, SLAMF8

## Abstract

Immune checkpoint inhibitors (ICI) for early-stage non-small cell lung cancer (NSCLC) have been approved to improve outcomes and reduce recurrence. Biomarkers for patient selection are needed. In this paper, we proposed an inflammasome-based risk score (IRS) system for prognosis and prediction of ICI response for early-stage NSCLC. Cox regression analysis was used to identify significant genes (from 141 core inflammasome genes) for overall survival (OS) in a microarray discovery cohort (*n* = 467). IRS was established and independently validated by other datasets (*n* = 1320). We evaluated the inflammasome signaling steps based on five gene sets, which were IL1B-, CASP-1-, IL18-, GSDMD-, and inflammasome-regulated genes. Gene set enrichment analysis, the Kaplan–Meier curve, receiver operator characteristic with area under curve (AUC) analysis, and advanced bioinformatic tools were used to confirm the ability of IRS in prognosis and classification of patients into ICI responders and non-responders. A 30-gene IRS was developed, and it indicated good risk stratification at 10-year OS (AUC = 0.726). Patients were stratified into high- and low-risk groups based on optimal cutoff points, and high-risk IRS had significantly poorer OS and relapse-free survival. In addition, the high-risk group was characterized by an inflamed immunophenotype and higher proportion of ICI responders. Furthermore, expression of *SLAMF8* was the key gene in IRS and indicated good correlation with biomarkers associated with immunotherapy. It could serve as a therapeutic target in the clinical setting of immunotherapy.

## 1. Introduction

Lung cancer, particularly non-small cell lung cancer (NSCLC), is the leading cause of cancer-related death in the United States and worldwide [1,2]. In early-stage NSCLC, 40–55% of these tumors recur despite surgery [3]. With the incorporation of cisplatin-based chemotherapy in the adjuvant setting for resected stage II to IIIA disease and in selected stage IB tumors, risk of recurrence or death is further reduced by 16% [4]. However, the percentage of patients with disease recurrence remains high after a median follow-up of approximately 5 years [5]. Osimertinib, a third-generation oral epidermal growth factor receptor (EGFR)-tyrosine kinase inhibitor (TKI), potently and selectively inhibits both EGFR-TKI sensitizing and EGFR p.Thr790Met resistance mutations and has been approved to be used in combination with adjuvant chemotherapy after it demonstrated significantly longer disease-free survival (DFS) than those who received placebo in a phase III randomized controlled trial (ADAURA) [6]. In North America, only about 10% of lung adenocarcinomas harbor EGFR mutation [7], therefore, the general population benefitting from this target therapy is inevitably restricted. As patients with stage I-III disease who relapse after surgery tend to have poor prognosis, identifying novel therapeutic approaches to reduce recurrence is a desperate need.

Immune checkpoint inhibitors (ICI) have revolutionized the treatment of advanced NSCLC with one-third of patients experiencing long-term survival [8]. Given the breakthrough results with PD-1 checkpoint inhibitors, there is a strong rationale to incorporate ICIs into the treatment of early-stage NSCLC as monotherapy or combination with chemotherapy. More recently, numerous phase I to III trials are investigating neoadjuvant and/or maintenance ICIs in early-stage NSCLC [9]. Preliminary data have indicated that adjuvant treatment with ICIs after adjuvant chemotherapy improves DFS and may play a critical role in reducing recurrence for resectable disease [10]. In contrast, as untreated tumors have a rich source of neoantigens, tumor-infiltrating lymphocytes, and an intact immune microenvironment, interest in the neoadjuvant setting is rising. Additionally, neoadjuvant single-agent PD-1 (nivolumab, pembrolizumab, or atezolizumab) in early-stage NSCLC has been proven to be safe and feasible, leading to major pathologic responses after at least two cycles of therapy [11,12].

In recent decades, inflammation has been recognized as one of the crucial events involved in cancer initiation, development, progression, angiogenesis, and invasion [13]. The mechanism behind it is orchestrated by inflammasome, a cytosolic multiprotein complex that is triggered by pathogen-associated molecules and cellular stress [14]. Inflammasome mediates the innate immune response and induces inflammatory programmed cell death, known as pyroptosis, through multiple reaction cascades involving the activation and release of proinflammatory cytokines IL1B and IL18 [15]. The inflammasomes are now considered cellular signaling hubs of the innate immunity that modulates inflammatory signaling and recruitment of immune cells to tumors. Interestingly, recent studies revealed contradictory results: both activation and inhibition of inflammasome signaling can reshape the tumor immunosuppressive environment and affect the efficacy of ICIs [16,17,18]. Therefore, systematical profiling of each inflammasome activation step is essential to clarify the differences in immune response.

In this study, we aimed to evaluate the inflammasome signaling steps based on five gene sets, which were IL1B-, CASP-1-, IL18-, GSDMD-, and inflammasome-regulated genes [19]. We identified that inflammasome genes play a significant role in the risk stratification of early-stage NSCLC. To the best of our knowledge, this is the first study to establish an inflammasome-based prognostic indicator for ICIs in early-stage NSCLC.

## 2. Materials and Methods

### 2.1. Microarray Data

Transcriptional data annotated with tissue types and clinical features for NSCLC were downloaded from the National Center for Biotechnology Information Gene Expression Omnibus (GEO, http://www.ncbi.nlm.nih.gov/geo, accessed on 4 January 2022). To minimize variations across platforms, the genomic profiles were based on the GPL 570 microarray platform (Affymetrix Human Genome U133 Plus 2.0 Array, HG-U133_Plus_2), and all the raw data were independently preprocessed with Robust Microarray Average normalization using the R/Bioconductor *oligo* package. The datasets were then merged after the batch effect was adjusted using the ComBat method derived from R/Bioconductor *sva* package [20]. 12 GEO datasets were retrieved with a total of 925 primary NSCLC tumors and 193 normal lung tissues.

### 2.2. RNA-Seq Data

Level-3 RNA-Seq data from patients with lung adenocarcinoma (LUAD) and lung squamous cell carcinoma (LUSC) were downloaded from the Cancer Genome Atlas (TCGA) using the bioinformatics tool Xena browser (https://xenabrowser.net/, accessed on 4 January 2022). The raw data were processed by expectancy maximization, and log2 transformed [21]. A final dataset was compiled of 1018 primary tumors and 110 normal lung tissues.

### 2.3. Construction of the Inflammasome Signature

Numerous genes were involved in inflammasome signaling, and their expression was mainly regulated by sensing (activated by inflammasome complexes), activation (activated by caspase-1), and termination (activated by GSDMD, IL1B, and IL18) in a sequential manner. Therefore, gene set analysis is necessary for biological interpretation and exploration [22]. The genes in each gene set were identified through literature review and meta-analyses, generating a total of 141 genes specific for the inflammasome signaling (Supplementary Table S1) [23].

### 2.4. Gene Set Enrichment Analysis (GSEA) and Differential Gene Analysis

Prior to constructing the risk score for clinical use, activity of the 141 inflammasome-related genes was first evaluated between lung tumors and normal tissues. The enrichment of a gene set, including all inflammasome-related genes in lung tumors, was analyzed using the GSEA function from the R/Bioconductor *clusterProfiler* package [24]. ES was derived by applying the weighted Kolmogorov−Smirnov statistic to a running sum of the ranked list. The ES was further normalized to account for the size of gene set input. False discovery rates (FDR) less than 0.001 were considered statistically significant. Differentially expressed genes (DEGs) were identified by a linear regression model using the R/Bioconductor *limma* package. DEGs with log2 fold change (FC) >1.5 and adjusted *p*-value < 1.0 × 10^−^^10^ were then mapped to the inflammasome gene set to assess the feasibility of inflammasome-based risk scores in both microarray and RNA-Seq platforms. To estimate the pathway activity for the five-inflammasome gene set, we used the single-sample GSEA (ssGSEA) function of the R/Bioconductor *GSVA* package to calculate the ssGSEA score of each tumor sample, which was min-max scaled. The pathway activity from the microarray and RNA-seq platforms was compared using hierarchical clustering of the normalized ssGSEA with the R library *ComplexHeatmap* package [25].

### 2.5. Construction of IRS

From the initial discovery set, 467 early-stage NSCLC patients who had complete survival information were used to derive IRS. The 141 core inflammasome genes were first filtered via the Cox regression model, and the genes with significant impact on survival were selected. Inflammasome-based risk score (IRS) was then calculated as a sum of multiplication of the expression level of each selected gene and its Cox regression coefficient. The IRS generated for each patient in the discovery set was visualized using R library *ComplexHeatmap* package. Surv_cutpoint function of the *Survminer* package was applied to stratify patients into high- and low-risk groups based on the most significant split according to the log-rank test.

### 2.6. Prognostic Value of IRS

To check whether IRS was prognostic for overall survival (OS) and relapse-free survival (RFS), three additional datasets were retrieved from the GEO repository for validation, which were GSE37745 (HG-U133_Plus_2), GSE30219 (HG-U133_Plus_2), and GSE68465 (HG-U133A). Manual curation was performed to select only stage IA to IIB patients with complete survival data and no adjuvant treatment. IRS was calculated in each validation dataset according to the Cox coefficients and patients were divided into high- and low-risk groups based on the optimal cutoff value.

### 2.7. Estimation of Immune Response

As we attempted to evaluate the ability of IRS in predicting the response to immunotherapy, a comprehensive tool was needed to obtain explicable results. TIDE, developed by Jiang et al., is a computational method used for forming a tumor immune evasion model (http://tide.dfci.harvard.edu/, accessed on 4 January 2022) [26]. In fact, the TIDE score has been demonstrated to be more effective and accurate than current methods in predicting the ICI response of melanoma and NSCLC patients. Through integrating the expression features of T cell dysfunction and T cell exclusion, potential regulators and indicators in ICI resistance can be estimated. Normalized gene expression profiles for patients without previous immunotherapy from the discovery set (467 samples) and GSE30219 (220 samples) were input. Predicted benefit from ICI was obtained with scores of TIDE, T cell dysfunction, and exclusion (reset threshold was set to zero). Furthermore, significant inflammasome genes for survival were also input as a custom biomarker and its predictive potential was assessed by area under curve (AUC) in various independent datasets with cancer types of melanoma, NSCLC, head and neck, and kidney cancers.

In additional to the TIDE scores, T cell-inflamed activity (TCIA) was applied to confirm the predicted efficacy of ICI [27]. TCIA was developed by Ayers et al. and included 18 genes for adaptive immune response. In our study, TCIA was quantified by calculating the ssGSEA score to assess the relationship with the IRS regarding efficacy of PD-1 blockade.

To decipher the difference of immune status according to the IRS risk groups, immune clusters in cancer and 62 immunomodulators covering Major Histocompatibility Complex (MHC) class genes, checkpoint stimulator genes and checkpoint inhibitor genes were included [28]. Furthermore, CIBERSORT was used to derive the relative infiltration level of 22 immune cell types in the microenvironment of the resected lung tumors using R script from its website (https://cibersort.stanford.edu/, accessed on 4 January 2022). Least Absolute Shrinkage and Selection Operator (LASSO) was used to estimate the coefficients for the predicted ICI response with penalty regularization. Inflammasome genes with non-zero coefficients were selected. Spearman coefficients were calculated for correlation among the selected genes and biomarkers associated with ICI response (Supplementary Table S2). Receiver operator characteristic (ROC) analysis was used to evaluate the discriminatory performance for the top influential genes compared with other common biomarkers for immunotherapy.

### 2.8. Survival Analysis

Time-dependent ROC and AUC were applied via R library *survivalROC* package (Kaplan−Meier, KM method) to evaluate the survival impact of IRS. According to the risk groups, both univariate and multivariate Cox proportional hazards regression and Kaplan–Meier (KM) survival analyses were performed using the R library *survival* package. In multivariate analyses, available clinical variables in each data set, such as smoking status, sex, age at diagnosis, histology, and tumor stage were adjusted.

### 2.9. Statistical Analysis

All statistical analyses were conducted in R software. Wilcoxon’s rank sum test was used to derive the *p*-value for continuous variables under defined conditions, and Fisher’s exact test was applied for categorical clinical features. In both tests, a *p*-value < 0.05 was considered statistically significant. For multiple testing in comparing gene expression and immune cell abundance between risk groups, FDR-adjusted *p*-value < 0.01 was considered significant.

## 3. Results

### 3.1. Feasibility of Core Inflammasome Genes in Early-Stage NSCLC

**Figure 1:**
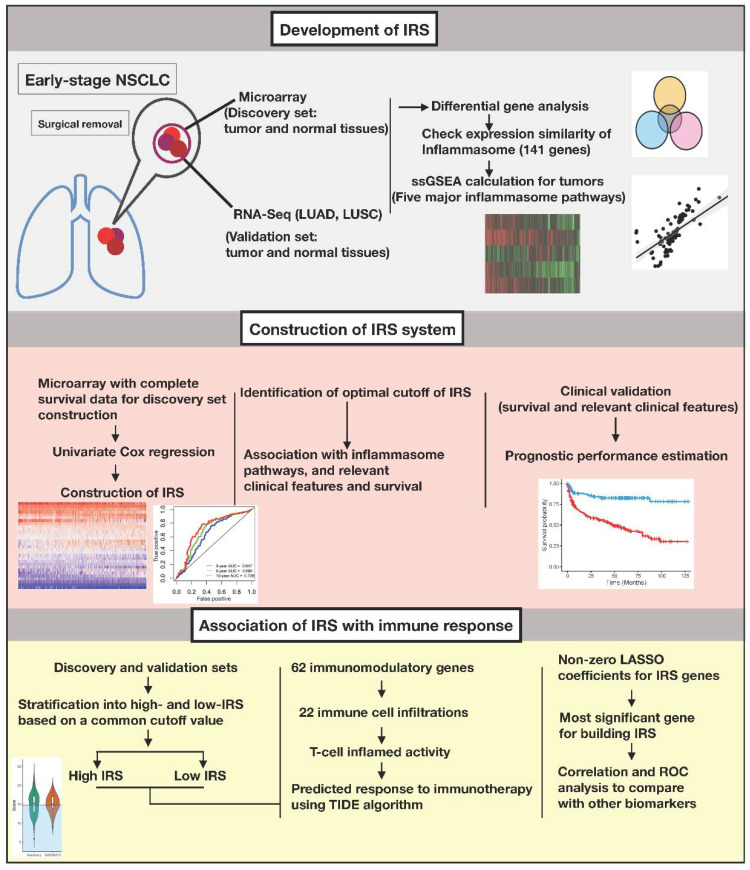
Schematic flowchart and bioinformatics workflow.

Genomic profiles from primary NSCLC tumors and normal lung tissues were obtained across different platforms. Eligible patients were more than 18 years old with traceable smoking habits, had stage I-II tumors, and had their tumors surgically removed without adjuvant therapy. Pathological types included adenocarcinoma, squamous cell carcinoma, large cell carcinoma, and adenosquamous carcinoma. The study workflow for our integrative bioinformatic analyses is summarized in Figure 1.

**Figure 2:**
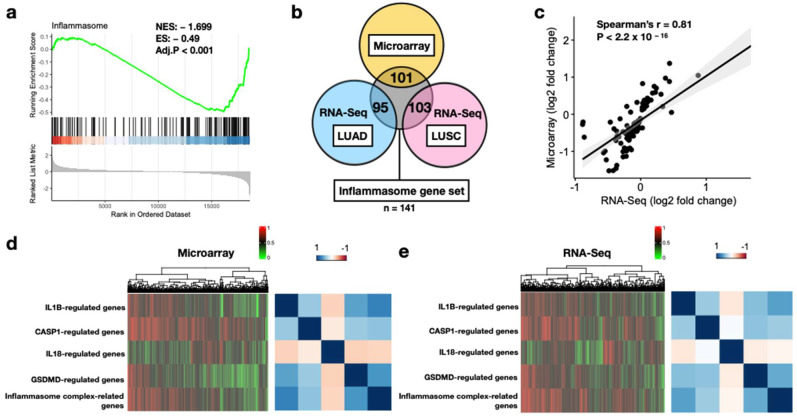
Feasibility of the core inflammasome genes in lung tumors. (**a**) GSEA plot of the gene set comprising all 138 core inflammasome genes in the microarray discovery cohort. Genes on the left (red) and right (blue) part of the graph correspond to high enrichment in NSCLC and normal tissues, respectively. The bottom plot (gray) depicts the value of the ranking metric as the computation goes down the list of ranked inflammasome genes. The normalized enrichment score (NES) and the false discovery rate-adjusted *p*-value (Adj.P) are displayed. (**b**) Venn diagram of differentially expressed genes related to inflammasome signaling among microarray and TCGA datasets. TCGA dataset includes lung adenocarcinoma (LUAD) and lung squamous carcinoma (LUSC). The numbers of genes overlapped are presented. (**c**) Spearman’s correlation of log2 fold change estimates from microarray and RNA-Seq platforms. (**d**) Hierarchical clustering of ssGSEA values for the five major inflammasome gene sets (**left**) and correlation matrix (**right**) in the microarray platform. (**e**) Hierarchical clustering of ssGSEA values for the five major inflammasome gene sets (**left**) and correlation matrix (**right**) in the RNA-Seq platform. Color bars indicate normalized ssGSEA value (**left upper**) and Spearman’s coefficient (**right upper**).

As we attempted to develop the discovery cohort from the microarray platform, applicability of the 141 core inflammasome genes in the resected lung tumors was first assessed. In Figure 2a, GSEA-generated results indicated significant enrichment scores in the tumors compared with the healthy tissues (normalized enrichment score [ES] = −1.699, *p* < 0.001). Furthermore, for DEGs identified between tumor and normal tissue samples, large overlaps were observed with the inflammasome genes (71.6%, 67.4%, and 73% for the microarray, LUAD, and LUSC, respectively, Figure 2b). In addition, log2 FC of gene expression values across sample types indicated high correlation between the microarray and RNA-Seq datasets (Spearman’s rho = 0.81, *p* < 2.2 × 10^−16^, Figure 2c). Hierarchical clustering and correlation matrix of the ssGSEA values for the five separate inflammasome gene sets indicated similar patterns in tumors from both datasets (Figure 2d,e). These results collectively indicated that the core inflammasome genes were aberrantly expressed and may play an important role in prognosis in early-stage NSCLC.

### 3.2. Construction of a 30-Gene IRS

**Figure 3:**
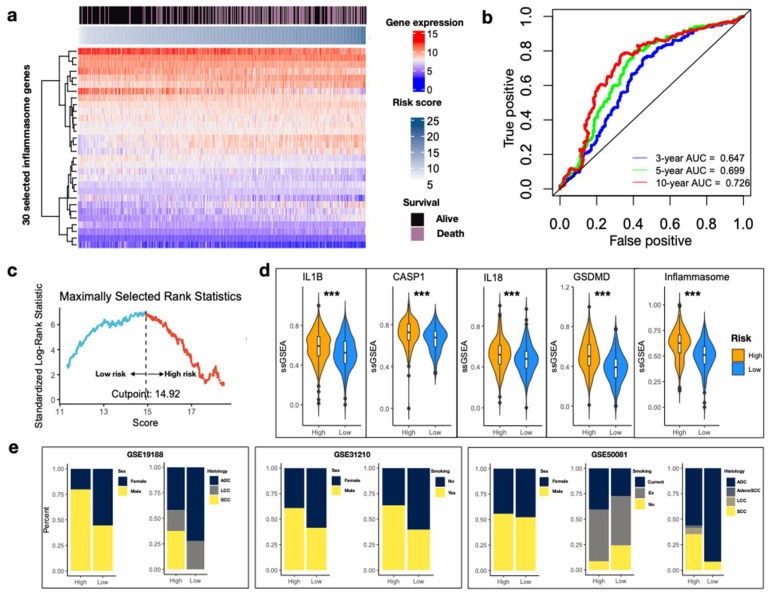
Development of the IRS. (**a**) Gene expression heatmap for inflammasome genes with survival impact in the discovery set. Columns are reordered by increasing IRS. Survival status is indicated above. Color bars indicate normalized gene expression and risk score. The heatmap was constructed using the R library *ComplexHeatmap* package [25]. (**b**) Time-dependent ROC curves at 3-, 5- and 10-year from the discovery set. (**c**) Standardized log-rank statistic to identify of the optimal cutoff value of IRS. The dashed line indicates the optimal split. IRS values above and below are colored in blue and red to indicate low- and high-risk, respectively. (**d**) Violin plots of ssGSEA values between high- and low-IRS for the five inflammasome gene sets. The ssGSEA values are normalized. Wilcoxon rank sum test *** *p* < 0.001. (**e**) Stacked bar plots indicating the proportion of samples in high- and low-risk IRS. Sample features include sex, histology, and history of smoking across three discovery datasets. Ex: previous smoking; ADC: adenocarcinoma; LCC: large cell carcinoma; AdenoSCC: adenosquamous cell carcinoma; SCC: squamous cell carcinoma.

Patients with complete survival information were extracted from the discovery set and 467 samples were subjected to univariate Cox regression analysis to identify the significant genes for survival. Cox coefficients of 30 inflammasome genes were then used to develop the IRS. The 30-gene expression profile of the discovery set was visualized in the heatmap with data sorted by increasing IRS (Figure 3a). Time-dependent ROC analyses indicated better discriminatory performance of the IRS at a 10-year survival than at 3- and 5-year, with an AUC of 0.726 (Figure 3b). Furthermore, an optimal cutoff value of 14.92 for risk grouping was selected using the log-rank statistics (Figure 3c).

### 3.3. IRS Is Associated with Inflammasome Activity and Various Clinical Features in NSCLC

In our discovery dataset of early-stage NSCLC, we further identified that IRS in the high-risk group corresponded to significantly higher ssGSEA of each biological step in inflammasome signaling (Figure 3d, all *p* < 0.001). Despite construction from survival analysis, this result indicated that IRS could represent the pathway activity as well. Male patients (GSE19188, *p* = 0.006345; GSE31210, *p* = 0.01025), habit of smoking (GSE31210, *p* = 0.0009994; GSE50081, *p* = 0.006347), and squamous histology (GSE19188, *p* = 0.002063; GSE50081, *p* = 7.865 × 10^−^^7^) were more likely to be enriched in the high-risk group, whereas adenocarcinoma accounted for a larger proportion in the group with low IRS (Figure 3e).

### 3.4. Prognostic Potential of IRS

**Figure 4:**
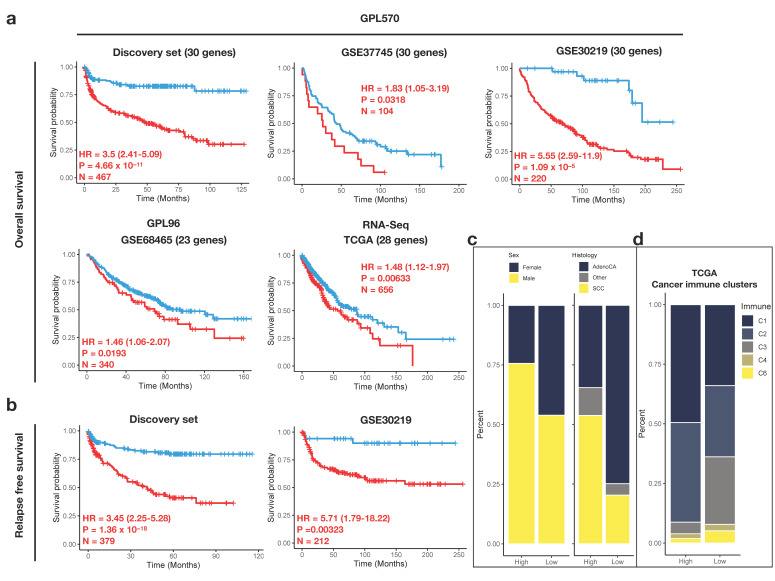
Risk-stratifying potential of the IRS gene signature. In each dataset, patients are stratified based on the optimal cutoff values for (**a**) OS and (**b**) RFS. KM survival analyses are performed and hazard ratio (HR), log-rank *p*-value, and the sample number are indicated on the graph. (**c**) Stacked bar plots indicating the proportion of samples in high- and low-risk IRS from the validation microarray platform. (**d**) Stacked bar plots indicating the proportion of samples in high- and low-risk IRS from the validation RNA-Seq platform. In TCGA validation cohort, proportion of cancer immune clusters (C1–C4 and C6) is illustrated.

The risk-stratifying potential of the 30-gene IFS signature for early-stage NSCLC was validated in additional four datasets. Patients with missing information of clinical factors, stage III-IV, and history of adjuvant systemic therapy or radiotherapy were manually removed. The final validation cohorts comprised a total of 1320 patients across different platforms (HG-U133_Plus_2: discovery set, GSE37745 and GSE30219, HG-U133A: GSE68465 and RNA-Seq: TCGA). In each cohort, the optimal cutoff value was determined separately, dividing the patients into high- and low-risk groups. Despite some missing inflammasome genes in GSE68465 and TCGA cohorts, IRS was remarkably associated with OS in all validation cohorts (Figure 4a), suggesting IRS was a strong prognostic indicator in the early-stage NSCLC patients. Additionally, RFS as another endpoint was selected, and it also demonstrated inferior outcomes in the high-risk group (discovery set and GSE30219, Figure 4b). Consistent with the cohorts in the discovery set, the high-risk group had a higher proportion of men (*p* = 1.946 × 10^−^^15^) and squamous histology (*p* < 2.2 × 10^−16^) (Figure 4c). Moreover, in the TCGA cohort, there were more C1 (wound-healing) immune clusters in the high-risk group and C3 (inflammatory) in the low-risk group, respectively (*p* = 0.0004998, Figure 4d). In support of this, the C3 cluster has been previously identified to be the most favorable outcome in a large cancer dataset [28].

To further confirm the prognostic ability of IRS, the multivariate Cox model was used to adjust for available clinicopathological variables such as age, sex, smoking history, tumor stage, and histology. IRS status remained significant in five microarray datasets, and two of them were validation cohorts (GSE30219 and GSE68465) (Supplementary Table S3). In TCGA, likely because of the lack of two inflammasome genes, the significance of IRS on survival was borderline (*p* = 0.07). In addition to IRS, early-stage patients with stage II lung cancer also had significantly poorer survival after adjustment (GSE31210, GSE50081, and TCGA). Nevertheless, our IRS signature indicated strong prognostic ability and even outperformed tumor stages (GSE31210: stage IA-II; GSE50081: stage IA-IIA) in the early-stage NSCLC patients.

### 3.5. Predict Response to ICI via IRS

**Figure 5:**
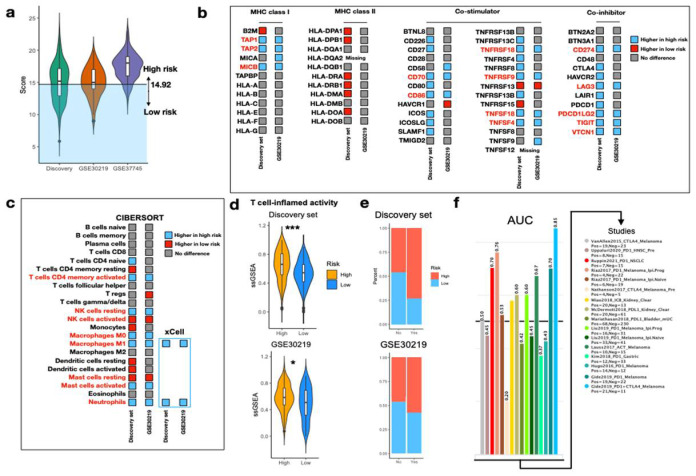
Evaluation of immunophenotypes between two risk groups. (**a**) Violin plot illustrating the IRS distribution on HG-U133_Plus_2 platform. A cutoff value of 14.92 is applied for patient stratification in the discovery set and GSE30219. (**b**) Expression of 62 immunomodulatory genes between high and low IRS. Genes with significantly high expression in the high- and low-IRS groups are colored in blue and red, respectively. Genes with no difference in expression are colored in gray. (**c**) Relative abundance of immune cells between high and low IRS using CIBERSORT. Immune cells with significantly high abundance in the high-risk group and low-risk group are colored in blue and red, respectively. Immune cells with no difference in abundance are colored in gray. Abundance of macrophages M1 and neutrophils is confirmed by xCell. (**d**) Violin plots portraying ssGSEA of TCIA between high- and low- IRS in the discovery set and GSE30219. Wilcoxon rank sum-test *** *p*  <  0.001, * *p*  <  0.05. (**e**) Stacked bar plots displaying the proportion of predicted responders to immunotherapy between risk groups in the discovery set and GSE30219. (**f**) AUC of IRS gene set as an input biomarker in TIDE to discriminate responders and non-responders across various datasets and cancer types.

To determine whether IRS was able to predict response to ICI, we applied the optimal cutoff value obtained from the discovery set to other validation cohorts also derived from HG-U133_Plus_2 (GSE30219 and GSE37745). With the exception of GSE37745, the discovery set and GSE30219 shared a common threshold (cutoff = 14.92) for significant risk stratification and were therefore used for further analyses (Figure 5a). As inflammasome is associated with innate immune response, we hypothesized the immune statuses between the two risk groups were different. In the panel of 62 immunomodulatory genes, we identified that the expressions of 14 genes (14/62) were remarkably higher in the high-risk group, compared with only one gene in the low-risk group (1/62) (Figure 5b). Among the 14 genes, the majority came from the co-stimulators, including *CD70*, *CD86*, *TNFRSF18*, *TNFRSF9*, *TNFSF18,* and *TNFSF4*. Interestingly, expression of genes critical for antigen presentation (*TAP1*, *TAP2,* and *MICB*) and ICI efficacy prediction (*CD274* and *PDCD1LG2*) were also elevated in the high-risk group. CIBERSORT was further applied to investigate relative infiltration of 22 immune cells between the two risk groups. We found tumors in the high-risk group comprised of richer activated CD4 memory T cells, resting NK cells, macrophages M0 and M1, activated mast cells, and neutrophils (Figure 5c). The relative abundance of macrophages M1 and neutrophils was validated by xCell. Furthermore, we found high-risk tumors were associated with significantly higher T cell-inflamed activity (TCIA), suggesting the presence of T cell-inflamed microenvironment (Figure 5d). In this case, a response to anti-PD1 blockade was expected [27]. Inferred response to immunotherapy via TIDE algorithm revealed higher proportion of responders in the high-risk group in both the discovery set (53.4% vs. 15.8%, *p* < 0.001) and GSE30219 (31% vs. 15.5%, *p* < 0.001) (Figure 5e). Using the 30 IRS genes as an input biomarker, the IRS genes achieved high predictive power in several melanoma (highest AUC = 0.85) and NSCLC (AUC = 0.7) (Figure 5f). Taken together, with the aid of IRS stratification system, response to ICI could be estimated in the early-stage NSCLC.

### 3.6. SLAMF8 Is the Key Inflammasome Gene for Predicting ICI Response

**Figure 6:**
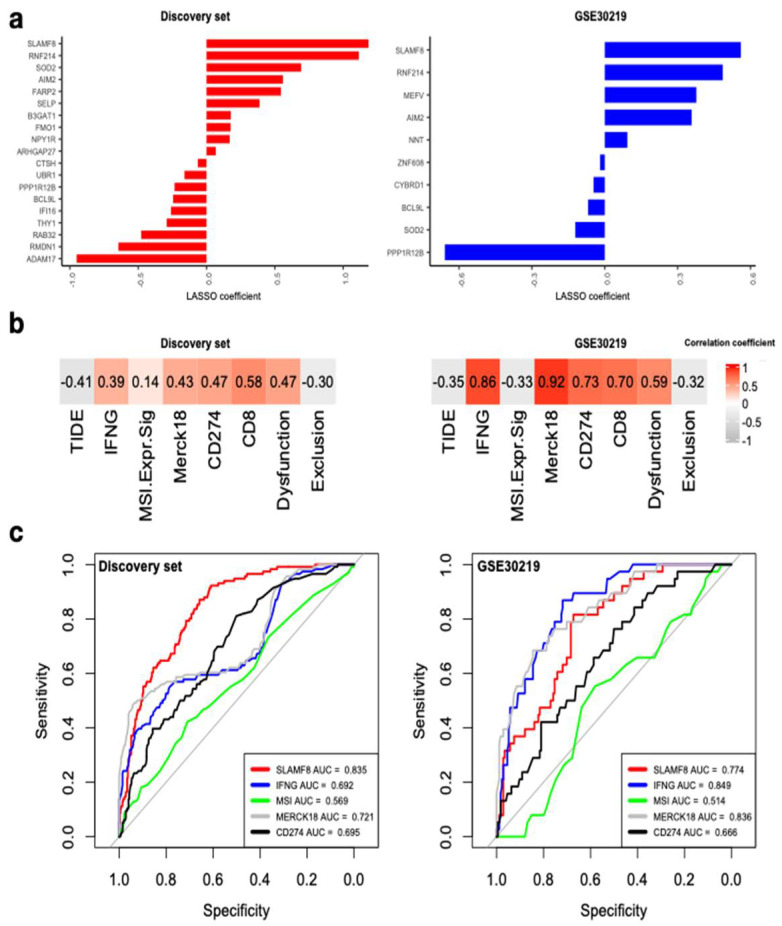
*SLAMF8* is the key IRS gene. (**a**) Genes with non-zero LASSO coefficients in the discovery set and GSE30219. (**b**) Correlation of *SLAMF8* expression with signatures associated with ICI response. (**c**) ROC curves for *SLAMF8* expression and other common biomarkers in discriminating the predicted responders and non-responders to ICI in the discovery set and GSE30219.

Using Least Absolute Shrinkage and Selection Operator (LASSO), we found *SLAMF8*, *RNF214*, *SOD2*, *AIM2*, *PPP1R12B*, and *BCL9L* were selected in both the discovery set and GSE30219 (Figure 6a). Among these genes, *SLAMF8* had the largest positive coefficients, suggesting a greater potential for discrimination. In both datasets, expression of *SLAMF8* was significantly high in the high-risk group (Figure S1). Correlation analysis demonstrated that *SLAMF8* had positive correlation with biomarkers of IFNG, Merck18, *CD274*, *CD8,* and dysfunction score; whereas negative correlation was discovered with TIDE and exclusion score (Figure 6b). Therefore, upregulation of *SLAMF8* correlated with positive ICI response. Further ROC analyses of response prediction to ICI revealed *SLAMF8* outperformed microsatellite instability and *CD274* in both datasets (Figure 6c). In the discovery set, *SLAMF8* had the highest AUC of 0.835. Overall, *SLAMF8* might serve as a target gene for immunotherapy in the early-stage NSCLC.

## 4. Discussion

In recent years, treatment focus of early-stage NSCLC has been shifted to neoadjuvant and adjuvant immunotherapy to overcome potential disease recurrence. At least 17 trials are currently ongoing to test the efficacy of ICIs in stages I-IIIB NSCLC [29]. The preliminary results were promising and a major pathological response of 20–80% could be achieved in the neoadjuvant setting. However, patient selection remains an unsolved issue and most trials adopted PD-L1 level as the molecular selection criteria. As NSCLC is an immunogenic tumor and a comprehensive biomarker, considering the immune microenvironment is an urgent need in the era of immunotherapy [30]. Recent evidence has revealed that activation of inflammasome signaling correlated with clinical outcome and orchestrated resistance to numerous cancer regimens [31,32]. Therefore, development of a tumor stratification system is necessary. For this reason, our study proposed the IRS that comprised 30 genes from a list of 141 core inflammasome genes. We discovered that IRS not only captured the pathway activity of the five core inflammasome signaling steps, but also had remarkable predictive value for prognosis. Further, patients with high IRS were identified to have richer immune cell infiltrations and a higher chance of response to ICIs.

Inflammasome signaling plays a vital role in both tumorigenesis and tumor immunosuppression [33]. The related pathways and interactions are complex and dual aspects of tumor promotion and suppression could be observed regarding different tumor stages and tumor microenvironments [33]. For example, inflammasome components such as Absent in melanoma 2 (*AIM2*) has been demonstrated to be aberrantly expressed in NSCLC, and its overexpression could lead to tumor growth [34,35]. Additionally, NLRP3 inflammasome activation enhances the proliferation and metastasis of the LUAD cell line [36]. Furthermore, IL1B is another key cytokine involved in inflammation and inflammasome activation; elevated levels of active IL1B contributed to intratumoral macrophage activation, accumulation of immunosuppressive myeloid cells, tumor growth, and lung cancer progression [37,38]. As inflammasome activation is a process involving multiprotein aggregation, single gene expression or protein-precursors may not necessarily reflect inflammasome activation. For this, our study explored the multidimensional profiling of inflammasome signaling to characterize the pathway activity of each inflammasome-regulated step. Additionally, we derived a 30-gene signature, IRS, for predicting the survival outcome. According to an optimal cutoff value (14.92), we identified significantly higher gene set scores (ssGSEA) in the high-risk IRS, indicating active signaling of the five major steps in early-stage NSCLC. Moreover, IRS was a strong prognostic indicator for OS and remained statistically significant after adjusting for age, sex, stage, or histology in the discovery and several validation datasets. IRS was also indicated to be predictive for RFS and could be incorporated into the clinical decision system to evaluate the risk of recurrence after surgery. Noteworthily, like external validation results for lung cancer patients, cancer stages had significant prognostic effect after adjustment [39]. Stage II NSCLC is characterized by larger tumor burden (tumor size and/or N1 nodal disease) and has been demonstrated to increase risk of death as compared with stage IA [39,40]. Our Cox regression results indicated poorer survival of stage II as referenced by stage IA NSCLC patients, which supported that staging was a reliable clinical factor that could be incorporated into decision-making systems in NSCLC. However, stage grouping is unable to play the role of evaluating both survival and immune response like IRS.

Despite the lack of complete IRS expression data in some validation datasets such as the TCGA RNA-Seq platform, the clinical utility remained strong. Intriguingly, high-risk IRS harbored more TCGA immune subtypes of C1 and C2, which together were characterized by high tumor proliferation, intratumoral heterogeneity and a higher proportion of M1 macrophages [28]; higher M1 macrophage proportion was validated by microarray platforms using CIBERSORT and xCell, in addition to richer neutrophils in the high-risk IRS (Figure 5c). M1 macrophage, different from its counterpart M2 macrophage, has been described as pro-inflammatory via secretion of many cytokines including IL1B, IL6, IL12, IL18, IL23, and TNF [41]. Inside this macrophage, cleaved active IL1B is produced by the action of the protease caspase-1, which is activated in the M1 macrophages by self-cleavage in the inflammasome complex [42]. Therefore, abundance of M1 macrophages in the high-risk IRS seemed to parallel active IL1B-, CASP1-, and inflammasome-complex-regulated pathways. Additionally, we observed a pro-tumor role of M1 macrophage in the early-stage NSCLC. Although the majority of evidence suggests an anti-tumor phenotype for the M1 subset, Oshi et al. identified that the transcriptomically defined M1 macrophages were associated with aggressive cancer biology (triple negative, advanced-stage and high-grade) and poorer survival in breast cancer [43]. With respect to the plasticity of macrophage polarization, our results may provide new insight about these antigen-presenting cells for the early-stage NSCLC, and we believed the intratumoral macrophages might play various and sometimes conflicting roles, depending on the clinical features, stages, histology, and the status of inflammasome signaling [44].

IL1B secreted by the M1 macrophages induces the expression of *PD-L1*, which impedes the cytotoxic T cells from directing the anti-tumor effect [45]. Other co-inhibitory genes such as *LAG3*, *PDCD1LG2*, *TIGIT,* and *VTCN1* upregulated in the high-risk IRS together shape the immunosuppressive phenotype [46,47,48]. Additionally, upregulated antigen-presenting machinery comprising *TAP1*, *TAP2,* and *MICB* suggested that the cytotoxic T cells could be selectively programmed against tumor cells. Ayers et al. revealed that interferon-γ-related gene profiling characterized the T cell-inflamed microenvironment, which in turn predicted clinical response to PD-1 blockade [27]. Here we demonstrated that early-stage tumors in the high-risk IRS had significantly higher TCIA and positively predicted ICI response via TIDE. Nevertheless, future clinical trials and experiments are needed to elucidate the complex interplay between inflammasome and immunotherapy. 

In a phase III trial of neoadjuvant PD-1 blockade followed by surgery in resectable NSCLC (CheckMate 816), researchers discovered that neoadjuvant nivolumab in addition to chemotherapy remarkably improved event-free survival and pathological complete response (24.0% vs. 2.2%), as compared with neoadjuvant chemotherapy alone [49]. Comparably, in IMpower010, atezolizumab provided after adjuvant chemotherapy in stage IB-IIIA NSCLC also significantly improved DFS [10]. In their intention-to-treat population, the hazard ratio (HR) was 0.81 (*p* = 0.04). Based on these two trials, immunotherapy has clear therapeutic benefits in early-stage NSCLC. Actually, as both clinical designs were not based on enrollment of pre-defined risk groups, there may be room for improvement with respect to immune response when biomarkers are considered [50]. At present, immunohistochemistry (IHC) of PD-L1 and tumor mutation burden (TMB) have been used to assess response to ICIs in NSCLC [51,52]. However, results of PD-L1 IHC are variable and often suffer from intratumoral or intertumoral heterogeneity [53]. In CheckMate 816, complete pathological response was not dependent on the PD-L1 expression level and TMB levels, indicating these two biomarkers are not feasible for predicting ICI responders under this circumstance. In our study, through using bioinformatic modeling, the threshold of IRS for risk grouping could be easily identified and its predictive value for both survival and ICI response could be estimated. Furthermore, IRS considers expression of 30 genes and could serve better prognostic purpose than single gene [54]. As high-risk IRS corresponded to higher chance of predicted ICI response, we believe IRS may add value in patient selection for ICI in the early-stage NSCLC in either neoadjuvant or adjuvant setting, probably with different thresholds. Nevertheless, further clinical studies are necessary.

Signaling lymphocytic activation molecule family 8 (SLAMF8) is the eighth member of SLAMF costimulatory receptors. SLAMF8 regulates development and function of various immune cells such as T lymphocytes, B cells, neutrophils, dendritic cells, macrophages, and eosinophils [55,56,57], and is reported to activate macrophages during inflammation. It has been demonstrated that *SLAMF8* expression was associated with T cell activation pathway, CD8 expression, and better response to anti-PD1 in gastrointestinal cancers [58]. Additionally, higher *SLAMF8* represented activation of antigen presentation and interferon-γ-mediated signaling in glioma [59]. Its association with NSCLC has never been elucidated. We identified *SLAMF8* expression had the strongest contribution to the IRS stratification system and correlated well with several immunotherapy-related biomarkers. Its positive correlation with T cell dysfunction indicated potential remolding of immune microenvironment with the use of ICIs. Taken together, *SLAMF8* expression could be a therapeutic target when immunotherapy is considered in early-stage NSCLC.

Most clinical trials of immunotherapy in NSCLC excluded patients with poor performance status (PS). Kartik Sehgal et al., however, has discovered an association between poor PS and significantly lower survival in advanced NSCLC patients receiving pembrolizumab monotherapy [60]. Of note, in this study, comorbidity score was not considered to be associated with disease control or survival. Conversely, in our study, only patients with resectable tumors were included. The relevant PS data in GSE37745 indicated that the PS of nearly 98% patients were ≤2. Moreover, the corresponding PS scores in TCGA dataset also revealed thar the majority of patients were PS ≤ 2. Therefore, in terms of the suitability for surgery, we believe the response to immunotherapy would not be affected to a significant degree, even though some case studies may help clarify this issue.

In this study, we utilized completely resected tumors as the source of genomic data, thereby minimizing the variations that might be encountered in biopsy specimens or bronchoscopy cytology. In this condition, tumor immunophenotypes may be properly characterized, providing valid results when immunotherapy is attempted. One of the limitations in our study is the lack of complete gene panel for calculating the IRS in several platforms, therefore limiting cross-platform compatibility. Although the ability of risk classification remained significant, demonstrating successful stratification of the early-stage NSCLC. Another limitation is that the ability of IRS in predicting ICI response was not validated by patients receiving immunotherapy. This is because the trials of ICIs in the early-stage patients are mostly ongoing, and the associated genomic data are not yet available. Moreover, as we only include resected and treatment naive tumors, whether neoadjuvant or adjuvant setting is better could not be concluded. Therefore, randomized controlled trials are needed to answer this question. Based on the inflammasome, we proposed the IRS and identified its association with survival, distinct immune microenvironment and ICI response. We believe this score system could assist in survival evaluation and the determination of potential ICI responder in early-stage NSCLC.

## Data Availability

Data have been described throughout the text and are available under accession codes GSE10245, GSE10445, GSE10799, GSE12667, GSE18842, GSE19188, GSE28571, GSE31210, GSE33356, GSE50081, GSE37745, GSE30219, GSE68465 from the National Center for Biotechnology Information Gene Expression Omnibus (GEO). Data from TCGA could be downloaded from Xena browser (https://xenabrowser.net/, accessed on 4 January 2022).

## Supplementary Materials

The following supporting information can be downloaded at: https://www.mdpi.com/article/10.3390/biomedicines10071539/s1, Figure S1: Violin plots showing SLAMF8 expression between high- and low-IRS in discovery set and GSE30219; Table S1: Inflammasome core genes; Table S2: Signatures associated with immune response; Table S3: Multivariate Cox models for IRS and other clinicopathological factors in different datasets.
