# Supplementary material for: Novel Inflammasome-Based Risk Score for Predicting Survival and Efficacy to Immunotherapy in Early-Stage Non-Small Cell Lung Cancer
Source: Biomedicines. 2022 Jun 28;10(7):1539. doi: 10.3390/biomedicines10071539 (PMC9313462; doi:10.3390/biomedicines10071539)
Supplement: Supplementary file 1 [file biomedicines-10-01539-s001.zip › biomedicines-1767818-supplementary.pdf]

Novel inflammasome-based risk score for predicting survival and efficacy to immunotherapy in early stage non-small cell lung cancer

Chih-Cheng Tsao, Hsin-Hung Wu, Ying-Fu Wang, Po-Chien Sheng, Wen-Ting Wu, Yun-Chen Huang, Yang-Hong Dai

Table S1. Inflammasome core genes.

Inflammasome genes	Symbol
CASP1_up_regulated_gene	TMEM260
CASP1_up_regulated_gene	TIFA
CASP1_up_regulated_gene	LSM4
CASP1_up_regulated_gene	CERS2
CASP1_up_regulated_gene	PLCG2
CASP1_up_regulated_gene	FAM219A
CASP1_up_regulated_gene	LAMTOR3
CASP1_up_regulated_gene	KLF11
CASP1_up_regulated_gene	CTSH
CASP1_up_regulated_gene	CAPZA2
CASP1_up_regulated_gene	ACTL6A
CASP1_up_regulated_gene	C10orf11
CASP1_up_regulated_gene	KLRB1
CASP1_down_regulated_gene	UBR1
CASP1_down_regulated_gene	PPP1R12B
CASP1_down_regulated_gene	ZNF512B
CASP1_down_regulated_gene	FARP2
CASP1_down_regulated_gene	PPIP5K1
CASP1_down_regulated_gene	PHKA1
CASP1_down_regulated_gene	BACE1
CASP1_down_regulated_gene	MRPS27
CASP1_down_regulated_gene	TRAPPC12
CASP1_down_regulated_gene	TAS1R3
CASP1_down_regulated_gene	CD247
CASP1_down_regulated_gene	CYBRD1

Inflammasome genes	Symbol
CASP1_down_regulated_gene	PCED1A
CASP1_down_regulated_gene	AP5M1
CASP1_down_regulated_gene	RMDN1
CASP1_down_regulated_gene	FBXO31
CASP1_down_regulated_gene	BLOC1S6
CASP1_down_regulated_gene	ATP10D
CASP1_down_regulated_gene	FMO1
CASP1_down_regulated_gene	FKBP5
CASP1_down_regulated_gene	NNT
IL1B_up_regulated_gene	ADAM17
IL1B_up_regulated_gene	SLC11A2
IL1B_up_regulated_gene	EREG
IL1B_up_regulated_gene	CSF1
IL1B_up_regulated_gene	ESR1
IL1B_up_regulated_gene	BID
IL1B_up_regulated_gene	BACH1
IL1B_up_regulated_gene	TNFRSF1B
IL1B_up_regulated_gene	MAPKAPK3
IL1B_up_regulated_gene	IFNAR2
IL1B_up_regulated_gene	NFKB2
IL1B_up_regulated_gene	OSMR
IL1B_up_regulated_gene	BTN1A1
IL1B_up_regulated_gene	IRF5
IL1B_up_regulated_gene	GNAQ
IL1B_up_regulated_gene	SULF2
IL1B_up_regulated_gene	SLC7A11
IL1B_up_regulated_gene	SLAMF1
IL1B_up_regulated_gene	SELP
IL1B_up_regulated_gene	NPY1R
IL1B_up_regulated_gene	PROKR1

Inflammasome genes	Symbol
IL1B_up_regulated_gene	SOD2
IL1B_up_regulated_gene	SERPINB2
IL1B_up_regulated_gene	RAB32
IL1B_up_regulated_gene	PTGER4
IL1B_up_regulated_gene	STX11
IL1B_up_regulated_gene	IGSF3
IL1B_up_regulated_gene	ARHGAP27
IL1B_up_regulated_gene	TBC1D9
IL1B_up_regulated_gene	TNIP1
IL1B_up_regulated_gene	SLAMF8
IL1B_up_regulated_gene	ACTR3B
IL1B_up_regulated_gene	PIM1
IL1B_up_regulated_gene	MAP3K8
IL1B_up_regulated_gene	POLR3K
IL1B_up_regulated_gene	BCL3
IL1B_up_regulated_gene	HOXD13
IL1B_up_regulated_gene	CDCA2
IL1B_up_regulated_gene	RND1
IL1B_up_regulated_gene	CERS5
IL1B_up_regulated_gene	GPR171
IL1B_up_regulated_gene	PTGS2
IL1B_up_regulated_gene	NFATC2
IL1B_up_regulated_gene	B3GAT1
IL1B_up_regulated_gene	CH25H
IL1B_up_regulated_gene	IL33
IL1B_up_regulated_gene	SLC5A1
IL1B_up_regulated_gene	NFKBIE
IL1B_up_regulated_gene	SOCS3
IL1B_up_regulated_gene	ELF3
IL1B_up_regulated_gene	SELE

Inflammasome genes	Symbol
IL1B_up_regulated_gene	TNFAIP2
IL1B_up_regulated_gene	TLR2
IL1B_up_regulated_gene	CHL1
IL1B_up_regulated_gene	CCL2
IL1B_up_regulated_gene	STEAP4
IL1B_up_regulated_gene	ZC3H12A
IL1B_up_regulated_gene	IL6
IL1B_up_regulated_gene	VCAM1
IL1B_up_regulated_gene	NFKBIZ
IL1B_up_regulated_gene	CXCL1
IL1B_up_regulated_gene	CCL20
IL1B_up_regulated_gene	LACC1
IL1B_up_regulated_gene	ZNF608
IL1B_up_regulated_gene	GBP6
IL1B_down_regulated_gene	LIM2
IL1B_down_regulated_gene	HS3ST2
IL1B_down_regulated_gene	COX5B
IL1B_down_regulated_gene	PDE4B
IL1B_down_regulated_gene	ASCL2
IL1B_down_regulated_gene	EHD2
IL1B_down_regulated_gene	SCN3A
IL18_up_regulated_gene	SC5D
IL18_up_regulated_gene	DYNC2H1
IL18_up_regulated_gene	BCL9L
IL18_up_regulated_gene	ALG9
IL18_up_regulated_gene	TMEM25
IL18_up_regulated_gene	NXPE4
IL18_down_regulated_gene	IFT46
IL18_down_regulated_gene	RNF214
GSDMD_up_regulated_gene	CD226

Inflammasome genes	Symbol
GSDMD_up_regulated_gene	KLRB1
GSDMD_up_regulated_gene	CXCR6
GSDMD_up_regulated_gene	THY1
GSDMD_up_regulated_gene	SAMD3
GSDMD_up_regulated_gene	TXK
GSDMD_up_regulated_gene	C5orf28
GSDMD_up_regulated_gene	FASLG
GSDMD_up_regulated_gene	RGS11
GSDMD_up_regulated_gene	CTSW
GSDMD_up_regulated_gene	MID1
GSDMD_down_regulated_gene	CAMK2B
GSDMD_down_regulated_gene	CNBD2
Inflammasome_complex_related_gene	NLRP1
Inflammasome_complex_related_gene	NLRP3
Inflammasome_complex_related_gene	CASP4
Inflammasome_complex_related_gene	CASP5
Inflammasome_complex_related_gene	NLRC5
Inflammasome_complex_related_gene	NLRP6
Inflammasome_complex_related_gene	NLRP12
Inflammasome_complex_related_gene	NLRP7
Inflammasome_complex_related_gene	NAIP
Inflammasome_complex_related_gene	NLRC4
Inflammasome_complex_related_gene	AIM2
Inflammasome_complex_related_gene	IFI16
Inflammasome_complex_related_gene	MEFV
Inflammasome_complex_related_gene	NLRP2
Inflammasome_complex_related_gene	PYCARD

Table S2. Signatures associated with immune response.

Signature	Description
IFNG	Interferon gamma (IFNγ) response biomarkers of 6 genes including *IFNG*, *STAT1*, *IDO1*, *CXCL10*, *CXCL9*, and *HLA-DRA*
CD278	*PD-L1* gene expression as the IHC surrogate
CD8	Infiltration of cytotoxic T lymphocyte (CTL), estimated from gene expression profile of *CD8A* + *CD8B*, is obtained from TIDE website
T cell dysfunction score	Phenotype score related to positive ICI response, is obtained from TIDE website
T cell exclusion score	Phenotype score related to negative ICI response, is obtained from TIDE website
MSI Expression Signature	Micro-satellite instability (MSI), a biomarker for positive ICI response, is obtained from TIDE website
Merck18	A biomarker for positive ICI response, is obtained from TIDE website

Table S3. Multivariate Cox models for IRS and other clinicopathological factors in different datasets.

GSE19188	HR	CI	P value
**IRS**			
**Low**	1		
**High**	4.65	1.61-13.4	0.004
**Sex**			
**Female**	1		
**Male**	1.93	0.94-3.99	0.07
**Histology**			
**ADC**	1		
**LCC**	0.77	0.35-1.67	0.51
**SCC**	0.66	0.34-1.28	0.22
**GSE31210**	HR	CI	P value
**IRS**			
**Low**	1		
**High**	4.36	1.69-11.25	0.002
**Sex**			
**Female**	1		
**Male**	1.31	0.63-2.74	0.47
**Stage**			
**IA**	1		
**IB**	1.4	0.52-3.8	0.51
**II**	3.07	1.21-7.78	0.018
**GSE50081**	HR	CI	P value
**IRS**			
**Low**	1		
**High**	2.17	1.17-4.04	0.013
**Sex**			
**Female**	1		

**Male**	1.99	1.12-3.53	0.018
**Histology**			
**ADC**	1		
**SCC**	0.39	0.20-0.78	0.007
**Smoking**			
**No**	1		
**Yes**	0.99	0.41-2.73	0.98
**Stage**			
**IA**	1		
**IB**	2.35	0.96-5.79	0.062
**IIA**	2.58	0.63-10.5	0.183
**IIB**	4.14	1.58-10.86	0.003
**Age**	1.03	0.98-1.08	0.268
**GSE37745**	HR	CI	P value
**IRS**			
**Low**	1		
**High**	1.03	0.54-1.95	0.93
**Sex**			
**Female**	1		
**Male**	1.02	0.66-1.57	0.94
**Histology**			
**ADC**	1		
**LCC**	0.76	0.37-1.54	0.44
**SCC**	0.92	0.55-1.55	0.75
**Age**	1.03	1.01-1.06	0.015
**GSE30219**	HR	CI	P value
**IRS**			
**Low**	1		
**High**	2.25	1.18-4.26	0.013

**Sex**			
**Female**	1		
**Male**	0.76	0.39-1.49	0.43
**Histology**			
**ADC**	1		
**LCC**	0.76	0.45-1.82	0.36
**SCC**	0.64	0.33-1.23	0.18
**Age**	1.04	1.01-1.07	0.0027
**GSE68465**	HR	CI	P value
**IRS**			
**Low**	1		
**High**	1.53	1.07-2.18	0.0192
**Sex**			
**Female**			
**Male**	1.46	0.73-1.72	0.606
**Smoking**			
**No**	1		
**Yes**	0.52	0.48-1.9	0.895
**Age**	1.05	1.02-1.07	< 0.001
**TCGA**	HR	CI	P value
**IRS**			
**Low**	1		
**High**	1.29	0.95-1.75	0.07
**Sex**			
**Female**			
**Male**	1.07	0.82-1.39	0.611
**Histology**			
**ADC**	1		
**SCC**	1.002	0.76-1.32	0.98

**Stage**			
**IA**	1		
**IB**	1.23	0.89-1.69	0.22
**IIA**	1.94	1.29-2.89	0.00125
**IIB**	1.68	1.18-2.38	0.00384
**Age**	1.02	1.0-1.03	0.016

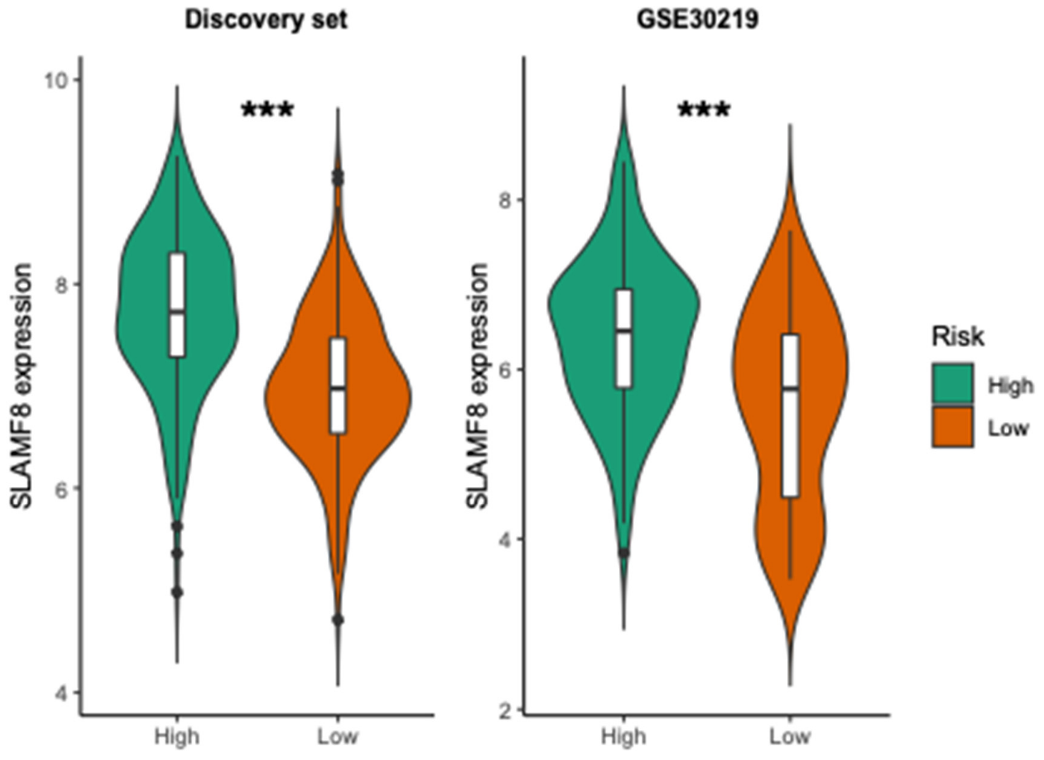

Figure S1. Violin plots showing SLAMF8 expression between high- and low-IRS in discovery set and GSE30219. Wilcoxon rank sum-test ***P < 0.001, **P < 0.01, *P < 0.05.
